# Inflaming and Immune-Resolving: The Ambivalent Role of Eosinophils in Osteoarthritis

**DOI:** 10.3390/ijms262210948

**Published:** 2025-11-12

**Authors:** Silvia Costantini, Paolo Dolzani, Veronica Panichi, Rosa Maria Borzì, Paulraj Balaji, Maria Daglia, Carla Renata Arciola

**Affiliations:** 1 Department of Medical and Surgical Sciences (DIMEC), Alma Mater Studiorum University of Bologna, 40136 Bologna, Italy; silvia.costantini6@unibo.it; 2 Laboratory of Immunorheumatology and Tissue Regeneration, IRCCS Istituto Ortopedico Rizzoli, 40136 Bologna, Italy; paolo.dolzani@ior.it (P.D.); rosamaria.borzi@icloud.com (R.M.B.); 3 PG and Research Centre in Biotechnology, MGR College, Hosur 635130, TN, India; balaji_paulraj@yahoo.com; 4 Department of Pharmacy, University of Naples Federico II, Via D. Montesano 49, 80131 Naples, Italy; maria.daglia@unina.it; 5 International Research Center for Food Nutrition and Safety, Jiangsu University, Zhenjiang 212013, China; 6 Laboratory of Immunorheumatology and Tissue Regeneration, Laboratory on Pathology of Implant Infections, IRCCS Istituto Ortopedico Rizzoli, 40136 Bologna, Italy

**Keywords:** osteoarthritis, eosinophils, low-grade inflammation, synovial inflammation, cartilage degradation, cytokines (IL-10; TGF-β), specialized pro-resolving mediators (SPMs), immune modulation, joint preservation, therapeutic targets in osteoarthritis

## Abstract

Osteoarthritis (OA), the most prevalent form of arthropathy, is characterized by progressive degradation of cartilage, synovial inflammation, and other pathological changes that gradually affect the entire joint. Once regarded as a purely degenerative disease with minimal immune involvement, recent evidence reveals that chronic low-grade inflammation, insidiously fueled by the destructive crosstalk between cartilage and synovium, plays a key role in OA pathophysiology. Among the immune cells involved, eosinophils have emerged as unexpected yet significant contributors, exhibiting both pro-inflammatory and immunoregulatory properties. Traditionally associated with allergic responses and antiparasitic defense, eosinophils can also secrete anti-inflammatory cytokines along with specialized pro-resolving lipid mediators (SPMs) that promote macrophage polarization toward reparative M2 phenotypes. Eosinophils may sustain inflammation or, conversely, act as “silent modulators” that subtly shape the immune microenvironment and support tissue homeostasis. This immunological plasticity positions them at the intersection of joint damage and repair. This article explores emerging evidence on eosinophil activity in OA, emphasizing their dual nature and potential as therapeutic targets to shift the joint *milieu* from a pro-inflammatory state toward resolution. Understanding eosinophil-mediated pathways may pave the way for novel strategies to reduce synovial inflammation, preserve cartilage integrity, and improve clinical outcomes.

## 1. Introduction

Osteoarthritis (OA) is the most prevalent form of arthritis, affecting nearly 500 million people worldwide. It represents a leading cause of disability among older adults, with its prevalence rising in parallel with aging populations and increasing obesity rates [1,2]. Hallmark pathological features include progressive cartilage erosion, synovial inflammation, subchondral bone remodeling, and osteophyte formation, all contributing to persistent pain, joint dysfunction, and reduced quality of life [3,4,5]. OA also imposes a substantial socioeconomic burden, driven by escalating healthcare costs and decreased productivity in aging societies [6].

Historically referred to as *osteoarthrosis*, OA was long considered a non-inflammatory, degenerative joint disease, primarily characterized by mechanical cartilage wear. This view clearly distinguished it from inflammatory arthritis, such as rheumatoid arthritis (RA) [7]. However, growing evidence has challenged this paradigm, revealing that chronic low-grade inflammation plays a central role in OA pathogenesis and progression, especially in later stages [8,9]. Pro-inflammatory cytokines such as IL-1β, TNF-α, and IL-6 drive the activation of matrix-degrading enzymes, most notably matrix metalloproteinases (MMPs) and aggrecans, that accelerate cartilage degradation and contribute to pain and stiffness [10]. Consequently, inflammation is now recognized as a key contributor to the progression of osteoarthritis, highlighting the need for therapeutic strategies that specifically target the underlying inflammatory pathways [11].

Among the various risk factors linked to OA [12] age is certainly the most relevant [13]. Twelve features, commonly known as hallmarks of aging, have been identified as the molecular framework leading to the progressive loss of cellular homeostasis and ultimately aging [14]. Since these traits are typical in all aging cells, their impact has raised particular interest in order to target age-associated diseases such as OA. Research has therefore been addressed to identify the molecular mechanisms responsible for their onset and progression. Chondrosenescence, defined as the age-associated decline in chondrocyte homeostasis, is a key contributor to the progression of OA [15]. With advancing age, there is a gradual loss of chondrocyte ability to maintain the delicate balance of anabolic and catabolic processes within the articular cartilage. The dysregulation of cellular processes, the impairment of autophagy, the accumulation of dysfunctional organelles and proteins, and the reduction in anabolic capacity collectively compromise extracellular matrix (ECM) integrity and impair tissue repair. Altered mechanotransduction has been demonstrated to reduce chondrocyte responsiveness to biomechanical cues, while disruption to intercellular communication has been shown to further exacerbate immune dysregulation and promote chronic low-grade inflammation, known as inflammaging [16]. In this regard, the concept of immunosenescence is also very relevant in OA; in fact, senescent immune cells (T and B lymphocytes and macrophages) in the nearby synovium foster inflammaging through the release of molecules belonging to the senescence-associated secretory phenotype (SASP) [17]. SASP is characterized by the release of pro-inflammatory cytokines and matrix degrading enzymes, which create a positive feedback loop that accelerates tissue degeneration and cartilage loss. These age-related changes initiate a sequence of events that lead to structural deterioration and amplify the inflammatory microenvironment, thus rendering the joint more susceptible to damage. In recent years, the urgent need for disease-modifying therapies for orphan diseases such as OA has driven the research into previously little explored realms. An interesting scenario has emerged around strategies aimed at exploiting the modulatory capacity exerted by immune cells in the joint microenvironment.

Among these, eosinophils, a type of granulocytic white blood cell, traditionally associated with responses to parasitic infections and allergic conditions such as asthma [18,19]. Known for releasing cytotoxic granules containing major basic protein (MBP) and eosinophil peroxidase (EPO), eosinophils were long regarded as purely pro-inflammatory cells capable of inducing tissue damage in allergic reactions.

However, recent findings have redefined this perspective. Eosinophils are now recognized as immune modulators with both pro-inflammatory and anti-inflammatory functions [20]. Beyond their classical roles, they contribute to the resolution of inflammation and maintenance of tissue homeostasis through the secretion of cytokines such as IL-10 and TGF-β, and lipid mediators like resolvins and protectins, which actively dampen immune responses [20,21].

This dual role, in both promoting and resolving inflammation—has sparked interest in their involvement in chronic inflammatory conditions, including OA [22]. Although eosinophils are less abundant than macrophages or neutrophils in OA-affected joints, they have been detected in synovial fluid and tissue from OA patients [23,24], suggesting a previously underappreciated regulatory function. Elucidating their contributions could provide valuable insights into OA pathogenesis and open new avenues for therapeutic intervention. If eosinophils exert anti-inflammatory effects in OA, enhancing this capacity may represent a novel disease-modifying strategy.

This article reviews emerging evidence on the role of eosinophils in OA, with a focus on their anti-inflammatory potential, the mediators they release to promote immune resolution, and their interactions with other immune cells within the OA joint microenvironment. Shedding light on these mechanisms, we aim to expand current understanding of immune regulation in OA and propose eosinophils as promising targets for therapeutic innovation.

## 2. Overview of Eosinophils in the Immune System

Although eosinophils constitute only 1–4% of circulating white blood cells in healthy individuals [25], they play essential roles in host defense and immune regulation.

These cells originate in the bone marrow from common granulocyte–monocyte progenitors, with their differentiation governed by a network of transcription factors, including GATA-1, C/EBPα, PU.1, and XBP1 [26].

Once mature, eosinophils circulate in the bloodstream for a brief period—typically between 3 and 24 h—before migrating into peripheral tissues [27]. Their recruitment is primarily directed by chemokines, notably eotaxin-1 (CCL11), which binds to the C-C chemokine receptor 3 (CCR3) expressed on the eosinophil surface [28]. Activation is mediated by cytokines such as interleukin-3 (IL-3), interleukin-5 (IL-5), and granulocyte–macrophage colony-stimulating factor (GM-CSF), with IL-5 being the most specific to eosinophil biology [29]. IL-5 promotes eosinophil maturation, facilitates their release into circulation, and extends their lifespan in peripheral tissues [30].

As granulocytic leukocytes, eosinophils are distinguished by large cytoplasmic crystalloid granules that store a wide array of preformed cationic cytotoxic proteins, including major basic proteins (MBP1, MBP2), eosinophil cationic protein (ECP), eosinophil-derived neurotoxin (EDN), and eosinophil peroxidase (EPO) [25]. Each eosinophil contains approximately 200 such granules, far fewer than the 2000 typically found in neutrophils, yet substantially larger in size. While neutrophils specialize in antibacterial defense, eosinophils primarily target multicellular parasites. Upon degranulation, these cytotoxic proteins are released and disrupt parasite membranes via electrostatic interactions [26].

Although these effector mechanisms are vital for parasite clearance, they can also exacerbate inflammation and promote tissue damage. Excessive eosinophil accumulation in peripheral organs—a condition termed eosinophilia—is associated with various diseases, including allergic asthma, atopic dermatitis, chronic rhinosinusitis, and eosinophilic gastrointestinal disorders such as eosinophilic esophagitis and inflammatory bowel disease [31]. In allergic responses, particularly in bronchial asthma, eosinophils release potent pro-inflammatory mediators like leukotrienes, which induce bronchoconstriction, and ciliostatic proteins that impair mucociliary clearance of the bronchial epithelium.

Recent evidence suggests that eosinophils are present in the synovial fluid and synovial membrane of osteoarthritis (OA) patients [32]. Boneva et al. observed that although eosinophils are rare in collagenase-induced OA models, they initially increase in the synovium following OA induction before subsequently declining, suggesting a dynamic role in OA pathogenesis [32].

The mechanisms that recruit eosinophils to synovial tissue in OA are not yet fully defined, but local inflammatory signals, such as chemokines, likely play a key role. Chemokines like eotaxin-1 (CCL11) and RANTES (Regulated upon Activation, Normal T cell Expressed and Secreted) are critical for guiding eosinophil migration to the sites of inflammation, particularly in chronic conditions [33]. CCL11 is central to eosinophil chemotaxis, as it binds to the CCR3 receptor on eosinophils, initiating signaling that directs their migration toward inflamed tissues. Eotaxin-1 is produced by several cell types, including epithelial cells, fibroblasts, and smooth muscle cells, especially in response to pro-inflammatory cytokines [34]. Similarly, RANTES recruits eosinophils to inflamed areas by binding to receptors such as CCR1, CCR3, and CCR5, and further activates eosinophils upon arrival [35]. RANTES is secreted by T cells, platelets, and endothelial cells and is often upregulated in chronic inflammatory diseases. In OA and other chronic joint diseases, these chemokine-driven mechanisms likely attract eosinophils to the synovium, where they may modulate inflammation and influence disease progression.

## 3. Dual Role of Eosinophils: Pro-Inflammatory and Anti-Inflammatory Functions

Recent research has increasingly highlighted the involvement of eosinophils in immune regulation, tissue remodeling, and homeostasis across various organ systems. These findings underscore their multifunctionality and challenge traditional paradigms by revealing their adaptability in both health and disease [19]. Lee et al. proposed the “LIAR hypothesis” (Local Immunity And/or Remodeling/Repair), which postulates that eosinophils are not randomly distributed throughout the body but preferentially localize to tissues characterized by high epithelial turnover, ongoing structural remodeling, or the presence of active stem cell niches. Within these dynamic microenvironments, eosinophil accumulation is supported by survival factors and mediators released during cell proliferation and tissue regeneration. The observed spatial distribution of these cells indicates the potential for eosinophils to function beyond their conventional effector capacities. This suggests that, in addition to their traditional role, they contribute to local immune surveillance, regulate tissue remodeling processes, promote repair mechanisms, and thereby maintain tissue integrity and homeostasis [36]. This immunomodulatory capacity is relevant for potential therapeutic purposes in OA. In fact, evidence from animal models shows that eosinophil-rich joints exhibit reduced inflammation, cartilage degradation, and bone erosion, whereas eosinophil depletion worsens joint damage [23].

In the early 1980s, based on density gradients, eosinophils were categorized into “normodense” and “hypodense” subtypes [37,38]. Hypodense eosinophils were found to display heightened activation, increased oxygen consumption, enhanced receptor expression, and greater cytotoxicity, features associated with severe disease and airway hyperresponsiveness. These findings suggest a potential role for eosinophils not only in disease progression, but also in broader aspects of immune regulation [39].

In murine models of asthma, two distinct lung eosinophil populations were identified: interstitial eosinophils (Siglec-F^med^CD11c^−^) and airway eosinophils (Siglec-F^high^CD11c^low^) [40]. These populations occupy distinct anatomical niches and respond differentially to allergic inflammation, demonstrating the phenotypic and functional diversity of eosinophils within a single organ. Building upon these observations, Mesnil et al. classified eosinophils into resident eosinophils (rEos) and inflammatory eosinophils (iEos) [41]. Located in the lung parenchyma, rEos help maintain tissue homeostasis through regulated degranulation and express CD62L, a marker of tissue residency. In contrast, iEos emerge following allergen exposure, lack CD62L, and express CD101, indicative of an activated phenotype. Transcriptomic analyses further revealed distinct functional profiles: rEos express genes that support homeostasis and suppress type 2 inflammation (e.g., Runx3, Anxa1), while iEos exhibit strong pro-inflammatory signatures, including elevated IL13Rα1, IL-6, and TLR4 expression [41].

Beyond the respiratory tract, eosinophils play critical roles in other tissues such as the gastrointestinal (GI) tract. In patients with inflammatory bowel disease (IBD), two distinct eosinophil subsets, active eosinophils (A-Eos) and basal eosinophils (B-Eos), have been identified [42]. A-Eos are enriched in co-stimulatory molecules (CD80, CD274) and pro-inflammatory mediators (IL-1β, TNF-α), suggesting a role in immune activation. Conversely, B-Eos express genes associated with tissue remodeling (e.g., MMP9, TGFB1) and appear to contribute to structural maintenance. Interestingly, these subsets may exist along a developmental continuum, with B-Eos differentiating into A-Eos in response to inflammatory stimuli [42].

**Figure 1:**
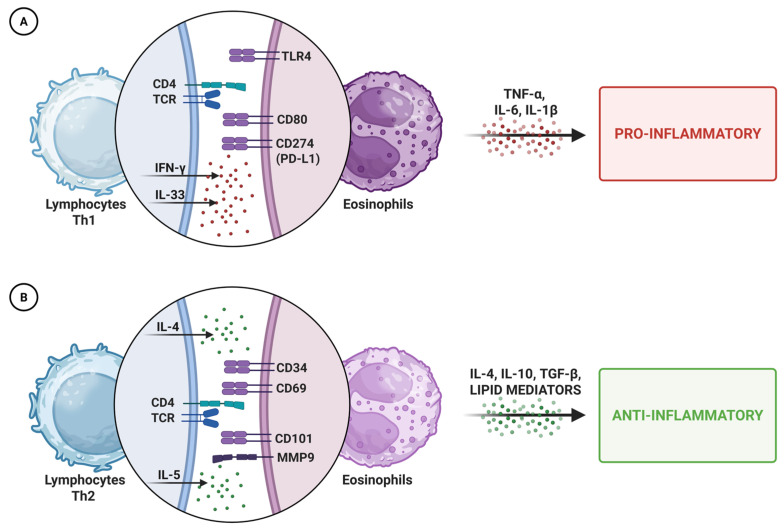
Schematic representation of eosinophil polarization. Depending on local cues, eosinophils can adopt a pro-inflammatory (**A**) or anti-inflammatory (**B**) phenotype, releasing distinct mediators that shape immune responses and tissue homeostasis. Abbreviations: Th1/Th2, T helper cell type 1/2; TCR, T cell receptor; TLR4, Toll-like receptor 4; IFN-γ, interferon-gamma; IL, interleukin; TNF-α, tumor necrosis factor-alpha; PD-L1, programmed death-ligand 1; TGF-β, transforming growth factor-beta; MMP9, matrix metallopeptidase 9.

One of the most intriguing aspects of eosinophil biology is their functional plasticity, which enables them to adapt to diverse immunological environments. Similarly to macrophages and neutrophils, eosinophils can polarize into type 1 or type 2 phenotypes, depending on local stimuli [43,44,45]. Type 1 eosinophils, activated by IFN-γ or bacterial products, adopt a pro-inflammatory profile, promoting inflammation and neutrophil recruitment. In contrast, type 2 eosinophils, induced by IL-4, exhibit anti-inflammatory and immunoregulatory properties. Polarization is driven by the surrounding cytokine milieu and environmental cues, enabling eosinophils to fulfill diverse roles across tissues and disease contexts (Figure 1) [46].

## 4. Eosinophil-Derived Anti-Inflammatory Mediators

Eosinophils possess a dual functionality within the immune system, enabling them to both initiate and resolve inflammation. As previously mentioned, this dual-faceted nature is mediated by the ability to secrete a wide range of signaling molecules. In pro-inflammatory contexts, eosinophils release cytokines such as IL-5, which promote inflammatory responses and recruit other immune cells to sites of infection or tissue damage [47]. Conversely, eosinophils also produce anti-inflammatory mediators, including IL-10, TGF-β, and specialized pro-resolving lipid mediators (SPMs), a class of bioactive lipids (e.g., resolvins, protectins, and maresins). These factors help counteract inflammation, facilitate its resolution, and restore immune homeostasis [18]. This broad and dynamic secretory profile underscores the remarkable versatility of eosinophils across different phases of immune responses, highlighting their context-dependent roles in both amplifying and resolving inflammation.

### 4.1. Interleukin-10

Eosinophils are well recognized as a source of IL-10, a potent anti-inflammatory cytokine essential for controlling and resolving inflammation [48]. IL-10’s primary role is to inhibit the production of key pro-inflammatory cytokines such as TNF-α, IL-1β, and IL-6, which are major contributors to tissue damage during immune responses [49,50]. By suppressing these cytokines, IL-10 helps reduce inflammatory intensity and limits tissue injury [51].

Beyond cytokine inhibition, IL-10 also modulates the activity of immune cells. It has been shown to downregulate macrophage and neutrophil activation, both critical players in sustaining inflammation [52]. contributing to a more regulated immune environment and supporting tissue homeostasis and protecting against chronic inflammatory disease progression [53].

### 4.2. Interleukin-4 (IL-4)

IL-4 released by eosinophils plays a key anti-inflammatory role, primarily by promoting the polarization of macrophages toward the M2 phenotype. This process has been observed in various tissues, including adipose tissue [54,55], bone [56], and the nervous system [57], contributing to the restoration of homeostasis [58].

Following injury, eosinophil-derived IL-4 facilitates regenerative processes such as hepatocyte proliferation [59], cardiac protection after myocardial infarction [60], and maintenance of anti-inflammatory responses in skin wounds [61]. Eosinophil depletion leads to reduced IL-4 levels, which in turn results in increased inflammation and excessive neutrophil infiltration. Administration of exogenous IL-4 restores an anti-inflammatory milieu [62].

### 4.3. Resolvins and Protectins

Eosinophils also actively produce lipid mediators involved in the resolution phase of inflammation, including resolvins, protectins, and maresins, collectively known as specialized pro-resolving mediators (SPMs) [63]. These molecules do not simply suppress inflammation but actively reprogram immune responses to promote resolution. SPMs reduce neutrophil infiltration, inhibit pro-inflammatory cytokine production, and promote efferocytosis, which is the process of clearing apoptotic cells, a fundamental mechanism for preventing chronic inflammation [64,65]. The eosinophilic capacity to produce SPMs highlights their importance in balancing immune responses in both acute and chronic settings [60,66,67,68].

Among SPMs, resolvins, derived from omega-3 fatty acids, are the most studied. They limit neutrophil accumulation at inflammation sites, reduce cytokine-mediated damage, and promote efficient clearance of apoptotic debris, preventing the shift from acute to chronic inflammation [69,70,71,72,73]. Protectins, especially Protectin D1 (PD1), act similarly [55,74].

### 4.4. Transforming Growth Factor-Beta (TGF-β)

Eosinophils are also a major source of TGF-β, a pleiotropic cytokine that regulates immune cell activity, tissue repair, and extracellular matrix remodeling [75]. TGF-β plays a context-dependent role, exerting either pro- or anti-inflammatory effects depending on the tissue type, the phase of the immune response, and local signaling cues [76,77,78].

Following tissue damage, TGF-β facilitates the resolution of inflammation by inducing regulatory T cells, which help prevent excessive immune responses and autoimmunity [79,80]. It also modulates extracellular matrix production, promoting structural stabilization during tissue repair [81].

## 5. Anti-Inflammatory Potential of Eosinophils in OA

In OA, where chronic inflammation persists within the joint, eosinophil-derived SPMs may be crucial in shifting the immune balance from a pro-inflammatory state toward inflammation resolution and help prevent the transition from acute to chronic inflammation [82,83,84]. Andreev et al. identified a novel subset of regulatory eosinophils in the joints expressing high levels of pro-resolving enzymes, such as 5-LOX and 12/15-LOX. These eosinophils facilitate inflammation resolution in chronic arthritis during remission stages of RA via the ILC2-IL-5 axis, supporting joint preservation and tissue regeneration [85].

Resolvins, reduce leukocyte infiltration into inflamed tissues [86], inhibit pro-inflammatory cytokine production [87] and enhance efferocytosis. These actions are particularly important in OA, where persistent infiltration of immune cells such as macrophages and T cells contribute directly to cartilage degradation and extracellular matrix breakdown, weakening joint integrity [88]. By producing resolvins, eosinophils help limit immune cell infiltration and reduce the inflammatory burden on joint tissues [89].

Another potential protective role of eosinophils in OA is their capacity to clear cellular debris and apoptotic cells from inflamed tissues. Accumulation of cellular debris in the synovial fluid is a hallmark of joint inflammation in OA, perpetuating immune activation by stimulating cytokine production and immune cell recruitment [90]. Eosinophil-derived IL-4 enhances efferocytosis, facilitating the engulfment and removal of apoptotic cells and cellular debris, thereby reducing the inflammatory load within the joint [91]. Dolitzky et al. further demonstrated that apoptotic cells induce an anti-inflammatory phenotype in eosinophils, decreasing their production of inflammatory cytokines [92]. This clearance function is crucial for maintaining tissue homeostasis and preventing chronic inflammation.

Chronic low-grade inflammation is now recognized as a major contributor to OA pathogenesis, playing a key role in joint degeneration and pain progression [93]. Unlike acute inflammation, which is a short-term protective response to injury or infection, chronic low-grade inflammation persists, resulting in the gradual degradation of cartilage and other joint tissues [94]. In OA, this inflammation is localized to the synovium and subchondral bone, where pro-inflammatory cytokines, immune cells, and damaged extracellular matrix components maintain a sustained inflammatory environment. A defining feature of chronic inflammation in OA is prolonged macrophage activation, which drives synovial inflammation through the release of pro-inflammatory cytokines such as IL-1β, TNF-α, and IL-6. These cytokines promote the expression of matrix metalloproteinases (MMPs) and aggrecans, which degrade cartilage extracellular matrix, leading to its progressive thinning [49].

Eosinophils play a significant role in modulating macrophage-driven inflammation, particularly by secreting anti-inflammatory cytokines like IL-10 [51]. Eosinophil-derived IL-10 directly inhibits pro-inflammatory macrophage activity, curbing the immune response within inflamed joints [95]. This modulation is particularly crucial in chronic inflammatory conditions like OA, where persistent macrophage activation contributes to tissue damage and disease progression [96]. Eosinophil-derived IL-10 promotes a phenotypic shift in macrophages from the pro-inflammatory M1 type to the anti-inflammatory M2 type [97]. IL-10 further influences other immune cells, contributing to an overall balanced immune environment; it inhibits neutrophil recruitment and activation and modulates T cell function, both of which are key contributors to synovial inflammation in OA [98,99].

Additionally, eosinophils, regulated by innate lymphoid cells type 2 (ILC2s) through IL-5 secretion, play a crucial role in reducing inflammation by releasing IL-4 [100]. This interaction is significant in inflammatory conditions like rheumatoid arthritis (RA) and collagen-induced arthritis (CIA), where the ILC2-eosinophil-IL-4 axis has a protective effect, correlating with decreased disease severity and underscoring IL-4’s role in modulating immune responses and controlling inflammation [101].

Recent research highlights the evolving role of regulatory eosinophils (rEos), identified as Siglec-F^int^, Ly6G^neg^, CD11b^+^, in resolving inflammation, particularly in RA. During RA remission, rEos are more prevalent, and their depletion can precipitate disease flares. Mechanistically, rEos promote anti-inflammatory M2 macrophage polarization through the ILC2-eosinophil-M2 axis, essential for inflammation control at joint tissues. This interaction between eosinophils and macrophages underscores the potential of eosinophils in resolving inflammation and preventing further tissue damage in OA [32].

In addition, eosinophils produce transforming growth factor-beta (TGF-β), which, in the context of OA, balances destructive and reparative processes within the joint, limiting excessive inflammation. Through their influence on immune responses and tissue repair pathways, eosinophils may play a dual role in controlling OA-associated inflammation. Beyond resolving inflammation, eosinophils promote the production of extracellular matrix components essential for cartilage health. Their regulation of proteins like S100A8 and S100A9 underscores their potential role in tissue repair [102].

**Figure 2:**
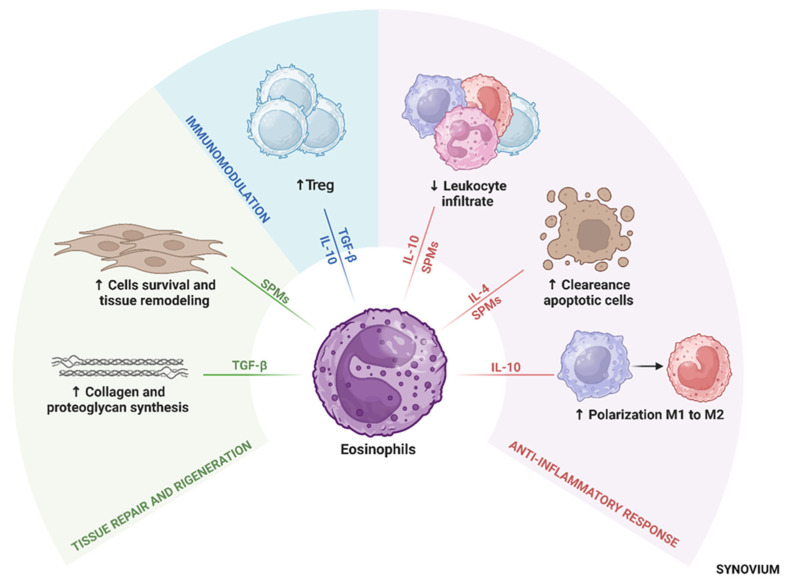
Central role of eosinophils in immune modulation in osteoarthritis. Eosinophils modulate inflammation, promote tissue repair, and support immune homeostasis by secreting anti-inflammatory cytokines, SPMs, and influencing macrophage polarization toward the M2 phenotype. These activities contribute to cartilage protection, resolution of inflammation, and joint tissue remodeling. Abbreviations: Treg, regulatory T cells; TGF-β, transforming growth factor-beta; IL, interleukin; SPMs, specialized pro-resolving mediators.

Eosinophil-derived TGF-β has been shown to regulate cartilage metabolism by stimulating the synthesis of proteoglycans and collagen, which are critical for maintaining cartilage structural integrity [103,104] and cartilage repair [105]. Thus, eosinophils help restore tissue homeostasis and support joint health in OA patients [16,103]. See (Figure 2). Pivotal concepts highlighting the anti-inflammatory role of eosinophils are summarized in Table 1.

## 6. Protective *Versus* Harmful Roles: Context-Dependent Effects

Although eosinophils have the potential to play a protective role in OA by resolving inflammation, their effects may be highly context dependent. Indeed, if their pro-inflammatory functions become dysregulated, eosinophils can contribute to tissue damage. For instance, although TGF-β can regulate inflammation in OA, its role is complex and warrants further investigation. Under certain conditions, elevated levels of TGF-β are closely associated with fibrosis and excessive tissue remodeling [106]. High concentrations of TGF-β can drive overproduction of extracellular matrix components, such as collagen and fibronectin, leading to thickening and stiffening of joint tissues [107]. This aberrant tissue growth and remodeling can result in the formation of fibrotic tissue within the joint, contributing to reduced mobility, joint stiffness, and ultimately, loss of function [108].

Several studies have highlighted the potential of helminth infections to reduce the severity of arthritis, with the anti-inflammatory cytokine IL-10 emerging as a key mediator of these protective effects [109,110]. In 2009, it was reported that infection with *Schistosoma mansoni* significantly attenuated disease in collagen-induced arthritis (CIA), a widely used model of autoimmune arthritis [111]. The protective effects were associated with decreased levels of pro-inflammatory cytokines such as IL-17A and TNF-α, and increased levels of IL-10. Similarly, Chen et al. showed that infection with *Nippostrongylus brasiliensis* resulted in Th2 cell and eosinophil accumulation in the joints, leading to reduced arthritis severity and protection from bone loss [112]. This effect was dependent on IL-4/IL-13-induced STAT6 signaling, with eosinophils contributing by promoting anti-inflammatory macrophage polarization within the joint.

However, the role of IL-10 in these mechanisms is complex. While IL-10 is essential for suppressing arthritis in certain experimental models, its necessity varies depending on the type of helminth and the model of immune-mediated disease. For example, protection from experimental autoimmune encephalomyelitis (EAE) induced by *Fasciola hepatica* infection occurred independently of IL-10 [113]. These findings suggest that while IL-10 is a central player in many helminth-induced anti-inflammatory responses, it is not universally required, and multiple immune-regulatory pathways are likely involved.

However, more research is needed to clarify under which conditions eosinophils exert anti-inflammatory effects in OA, and what factors modulate their function within the synovial microenvironment. Understanding the diverse and sometimes paradoxical roles of eosinophils in joint inflammation may provide new insights into therapeutic strategies aimed at harnessing their protective properties, potentially improving outcomes for patients with OA.

For their subtle regulation of immune homeostasis and responsiveness, eosinophils may be conceptualized as ‘silent modulators’ within the immune system. This concept, translated from epigenetic and pharmacological mechanisms [114,115], refers, beyond their well-known effector functions, to the essential roles eosinophils exert in the fine-tuning of tissue homeostasis without triggering overt immune activation.

Eosinophils are increasingly recognized for their regulatory role in maintaining immune balance, even within joints and bone. Recent findings suggest their involvement in the resolution of inflammation and local immune modulation. We will now recall some key points. Eosinophils act as tissue-resident immunomodulators. Indeed, beyond their known roles in immune defense, eosinophils reside in various healthy tissues, such as the spleen, gastrointestinal tract, thymus, adipose tissue, and uterus, and are increasingly seen as critical players in sustaining tissue equilibrium. In healthy tissues—most notably in the gastrointestinal tract—eosinophils contribute to tissue maintenance and immunological balance through non-inflammatory, constitutive activity. In the joint tissues, eosinophils appear to influence local immune tone subtly, participating in cellular crosstalk and promoting resolution over escalation. Thus, they help shape a homeostatic microenvironment [116]. This emerging perspective highlights their significance in sustaining long-term tissue equilibrium, particularly under chronic or subclinical conditions.

Recent studies have shown that eosinophils play a protective role in bone homeostasis. In murine models, the absence of eosinophils led to decreased bone mass and exacerbated bone loss in conditions such as hormone deficiency and inflammatory arthritis. Conversely, increasing eosinophil numbers improved bone density and conferred protection against both hormonally- and inflammation-induced bone loss. This effect is mediated by eosinophil peroxidase, which suppresses the generation of reactive oxygen species (ROS) and inhibits MAPK signaling pathways in osteoclast precursors, thereby reducing bone resorption [106].

Notably, the absence of eosinophils was associated with a reduced presence of M2 macrophages in both healthy and arthritic joints, underscoring their role in immune modulation even in non-inflammatory conditions [85].

## 7. Potential for Therapeutic Modulation

As the understanding of the roles of eosinophils in immune regulation and inflammation resolution has evolved, their potential as therapeutic targets in OA has gained attention.

While traditional OA treatments primarily focus on pain relief and symptom management, targeting the molecular mechanisms driving chronic inflammation could lead to more effective, sustained therapeutic outcomes. For instance, drugs or biologics that increase specialized pro-resolving mediator (SPM) levels in OA joints have shown promise in limiting the chronic low-grade inflammation characteristic of the disease and alleviating pain [117]. Resolvin D1 liposomes, in particular, have demonstrated efficacy in preclinical OA models [117].

In clinical studies, SPMs such as 17-HDHA have been associated with reduced pain and improved quality of life in OA patients [118,119,120]. Moreover, nanoliposomal formulations of Resolvin D1 (Lipo-RvD1) promote anti-inflammatory M2 macrophage polarization and show therapeutic potential in OA [121].

Direct injections of TGF-β into degenerating intervertebral disc tissue in vitro significantly promoted proteoglycan synthesis and reduced tissue resorption by decreasing MMP-2 secretion, demonstrating tissue-protective effects [122]. Eosinophil-derived TGF-β might have similar regenerative effects, suggesting a role in tissue repair [123].

Recent work by Lee et al. demonstrated that TissueGene-C (TG-C), a novel cell and gene therapy for OA, increased IL-10 and TGF-β1 levels, promoting M2 macrophage polarization by inducing arginase 1 expression and reducing the M1 macrophage marker CD86 expression [124].

Another potential therapeutic strategy involves IL-5, which plays a critical role in eosinophil growth, differentiation, and activation [125]. Therapies targeting the IL-5 pathway, such as Mepolizumab and Benralizumab, are currently used to treat eosinophilic asthma and other eosinophil-related disorders [126]. These drugs reduce circulating eosinophils or inhibit their activation, thereby mitigating excessive eosinophilic inflammation [126]. However, concerns have arisen over a minimal but potential risk of developing OA or other inflammatory joint conditions, although clear evidence linking these therapies directly to these conditions is lacking [127]. Similarly, Dupilumab, an anti-IL-4/-13 receptor biologic used in atopic dermatitis, has been associated with joint manifestations such as seronegative arthritis and enthesopathy [128].

While eosinophil-targeted therapies hold promise as a novel approach to treating OA, they may be most effective when combined with existing treatments. For example, IL-4, when combined with prednisolone, plays a crucial role in preventing cartilage and bone degradation by inhibiting inflammation-driven processes such as MMP-1 activity and preserving chondrocyte integrity [129,130]; when combining with osteoprotegerin has yielded promising results by significantly improving bone mineral density, reducing bone resorption markers by up to 68%, and preventing bone loss through the inhibition of pro-inflammatory cytokines like IFN-γ [131,132].

Emerging regenerative strategies, such as mesenchymal stem cell (MSC) therapies, may also benefit from the integration of eosinophil-targeted modulation. While primarily investigated in inflammatory arthritis models, recent preclinical studies have shown that MSCs combined with interleukin-4 (IL-4) can reduce inflammation and preserve cartilage integrity. In a murine model of rheumatoid arthritis, Haikal et al. [133] demonstrated that IL-4-enhanced MSCs attenuate joint damage by downregulating pro-inflammatory cytokines (e.g., TNF-α, MCP-1), reducing autoantibody levels, and protecting cartilage from structural erosion. Although eosinophils were not directly examined, IL-4 is a key mediator of type 2 immunity and is known to modulate eosinophil recruitment and activation. This raises the intriguing possibility that future OA therapies might harness eosinophil-linked immunoregulatory circuits, potentially through IL-4-enriched MSCs, to simultaneously support immune resolution and tissue regeneration.

**Figure 3:**
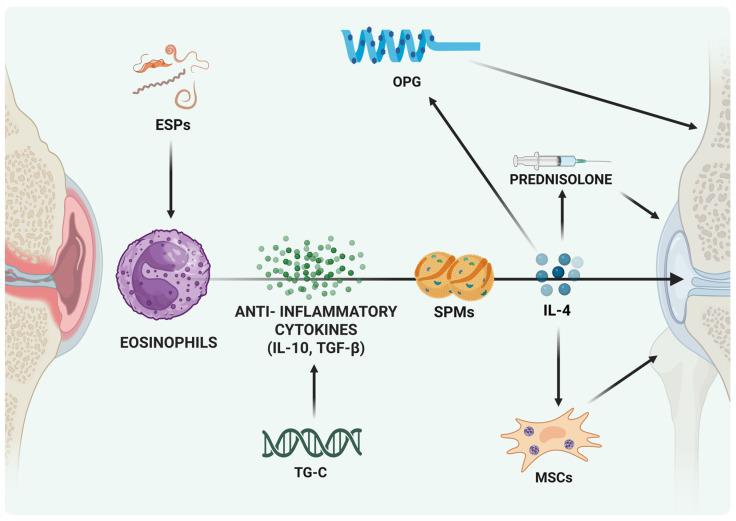
Eosinophil-Mediated Protective Strategies in Joint Disease. Different experimental approaches aimed at fostering the effects of mediators released by eosinophils and support their protective role in joint disease. Abbreviations: ESPs, excretory/secretory products; MSCs, mesenchymal stem cells; IL-4/IL-10, interleukin-4/10; SPMs, specialized pro-resolving mediators; TGF-β, transforming growth factor-β; TG-C, genetically modified chondrocytes; OPG, osteoprotegerin.

Despite the therapeutic promise of eosinophil-targeted interventions, several challenges must be addressed before their safe and effective implementation in osteoarthritis (OA) treatment. A key limitation lies in the context-dependent nature of eosinophil activity. In diseases such as asthma and eosinophilic esophagitis, eosinophils are well-documented drivers of excessive inflammation and tissue damage [134,135]. This duality underscores the need for precise modulation strategies that enhance eosinophils’ anti-inflammatory and pro-resolving functions without triggering their pathogenic potential. Achieving this balance will be critical to translating eosinophil-based approaches into viable clinical therapies for OA (Figure 3). Unfortunately, despite various clinical trials targeting eosinophil, to date there are no ongoing trials harnessing the anti-inflammatory potential in musculoskeletal diseases.

Emerging evidence highlights the potential of helminth-derived products to induce *trained immunity* in innate immune cells, offering novel therapeutic avenues for chronic inflammatory diseases such as osteoarthritis (OA).

A very recent review by Carrera Silva et al. [136] illustrated how helminth excretory/secretory products (ESPs) can epigenetically and metabolically reprogram innate immune cells, promoting durable anti-inflammatory responses and tissue homeostasis. This helminth-trained immunity paradigm provides a mechanistic basis for the long-observed immunomodulatory effects of helminth infections.

In this connection, experimental data reveal that ESPs from *Trichuris suis* induce a trained anti-inflammatory phenotype in bone marrow-derived macrophages, limiting inflammatory responses and suggesting therapeutic potential in autoimmune and inflammatory conditions [137]. Similarly, a peptide derived from the trematode *Fasciola hepatica* was shown to reverse the trained pro-inflammatory phenotype of macrophages in murine models, indicating a capacity to modulate innate immune memory toward resolution of inflammation [138]. Furthermore, the excretory/secretory product ES-62 from the filarial nematode *Acanthocheilonema viteae* demonstrated protection against collagen-induced arthritis, potentially linking gut-bone marrow crosstalk with modulation of innate immunity [139].

IL-4 and other cytokines produced during helminth-driven immune responses can modulate eosinophil recruitment and activation, potentially contributing to the anti-inflammatory and tissue reparative effects observed in helminth-induced trained immunity. Thus, integrating eosinophil biology with helminth-mediated immune modulation may enhance therapeutic strategies aimed at resolving chronic inflammation and promoting joint tissue regeneration in OA.

Translating these insights into clinical therapies will require optimization of ESP delivery methods, dosing, and safety profiles. Synthetic exosome mimetics and targeted delivery systems represent promising tools to overcome current challenges and enhance the therapeutic potential of helminth-based interventions.

In summary, the concept of helminth-trained immunity opens exciting prospects for innovative OA treatments by integrating immune regulation with tissue regeneration.

Various phytochemicals, including quercetin, curcumin, resveratrol, and β-boswellic acid, exhibit multifaceted anti-inflammatory and immunomodulatory effects [140,141]. By inhibiting NF-κB, NLRP3 inflammasome, and TLR4/IL-1R signaling, they could indirectly support eosinophil-mediated resolution [140].

Both curcumin [141] and resveratrol [142] suppress IL-1β-induced injury of articular chondrocytes by inhibiting the NF-κB pathway and NLRP3 inflammasome activation, favoring chondrocyte survival and reducing inflammation [142,143].

In a murine model of destabilization of the medial meniscus, β-boswellic acid (BBA) significantly reduced cartilage degradation and synovitis. In vitro, BBA inhibited IL-1β and TLR4-mediated induction of inflammatory mediators in OA synovial tissues [144].

These natural agents offer an interesting therapeutic avenue to rebalance immune responses and slow OA progression. The inhibition of key inflammatory pathways suggests a possible indirect pro-resolving role, compatible with that of eosinophils (rEos).

Despite the recent development in eosinophils related models in vitro and in vivo, currently the further broadening of research in this field is impaired by unresolved experimental restraints. Firstly, human eosinophils are short-lived, present at low abundance in circulation, and technically challenging to isolate and culture [145,146,147], thus, case by case, precautions must be taken to set up reliable in vitro models. Relevant differences emerge also between human and animal eosinophils [148]. For example, murine eosinophils lack certain IgE receptors and display host dependent differences in metabolic interactions with other cells, calling for attention to translational fidelity [149,150]. Furthermore, current models gloss on the complexity to reproduce tissue-specific interactions and homeostatic roles yet often induce compensatory changes that confound interpretation [151,152,153]. Moreover, depletion models such as eosinophil-deficient mice reveal broad functional outcomes but also highlight the challenges in reliably distinguishing stage-specific or context-dependent effects [151,154]. Consequently, extrapolating mechanistic insights from existing models to human eosinophil behavior requires caution. Integrated approaches combining humanized mouse models, organoid systems, and single-cell analyses are increasingly necessary to capture eosinophil heterogeneity and context-dependent regulatory (“silent modulatory”) functions.

## 8. Conclusions

Eosinophils, once viewed as pro-inflammatory cells, are now increasingly recognized as versatile regulators of immune homeostasis. By releasing both pro- and anti-inflammatory mediators, they can either promote resolution or sustain low-grade chronic inflammation, making them a double-edged sword in osteoarthritis immunopathology. While their immunomodulatory potential opens promising therapeutic possibilities, their context-dependent behavior, reflecting a complexity still being unraveled, calls for further investigation and optimization of integrated and reliable experimental models in the pursuit of effective treatments for the chronic and debilitating osteoarthritic disease.

## Figures and Tables

**Table 1:** Relevant anti-inflammatory mediators of Eosinophils (Eo).

Tissue/Cells	Mediators Involved	Effects Observed	Experimental Model	Ref.
Fat tissues	IL-4	Eo are the major IL-4-expressing	Murine	[54,58]
Liver	IL-4	Eo promote proliferation of quiescent hepatocytes	Murine	[59]
Cell lines	IL-4	Eo support resolution of inflammation	Murine	[62]
Cell lines	IL-4/IL-13	Decrease kinin receptors in a STAT6-dependent mechanism	Human/Murine	[56]
Dermis	IL-4/CCL24	Eo maintain dermis-resident macrophages as replicative niches for *Leishmania major*	Murine	[61]
Myocardium	IL-4/Cationic protein mEar1	IL-4 establishes a cardioprotective role of Eo in post-MI hearts	Humans/Murine	[60]
Blood	SPMs, Resolvin D1/D2/E2	Resolution of acute injury and bacterial infection	Human PBMCs	[72]
Peritoneum	SPMs/Protectin D1	Resolution of acute peritonitis	Murine	[55,66]
Lung	Resolvin D1	Resolution of LPS-induced lung injury	Murine	[71]
Synovium	Resolvin D1	RvD1 inhibited OA-FLS proliferation and reduced MMP-13 and IL-1β secretion	OA patients	[74]
Colon	Chemokines CXCL1/CXCL2 Protectin D1	Eo exert a protective effect via production of anti-inflammatory lipid mediators	Murine	[69]
Lung/Colon	TGF-β	TGF-β signaling regulates eosinophil behavior	Murine	[75]

## Data Availability

No new data was created in this study. Data conceptualization is contained within the article.
